# Quiet Sleep Organization of Very Preterm Infants Is Correlated With Postnatal Maturation

**DOI:** 10.3389/fped.2020.559658

**Published:** 2020-09-22

**Authors:** Léa Cailleau, Raphaël Weber, Sandie Cabon, Cyril Flamant, Jean-Michel Roué, Géraldine Favrais, Géraldine Gascoin, Aurore Thollot, Maxime Esvan, Fabienne Porée, Patrick Pladys

**Affiliations:** ^1^Department of Neonatology, University Hospital of Rennes, Rennes, France; ^2^Univ Rennes, CHU Rennes, INSERM, LTSI - UMR 1099, Rennes, France; ^3^Department of Neonatology, University Hospital of Nantes, Nantes, France; ^4^Department of Neonatology, University Hospital of Brest, Brest, France; ^5^Department of Neonatology, University Hospital of Tours, Tours, France; ^6^Department of Neonatology, University Hospital of Angers, Angers, France; ^7^Department of Neonatology, University Hospital of Poitiers, Poitiers, France; ^8^Univ Rennes, CHU Rennes, Inserm, CIC 1414 (Center d'Investigation Clinique de Rennes), Rennes, France

**Keywords:** preterm infant, sleep, maturation, visualization software, annotation software, video

## Abstract

**Background:** Sleep is an important determinant of brain development in preterm infants. Its temporal organization varies with gestational age (GA) and post-menstrual age (PMA) but little is known about how sleep develops in very preterm infants. The objective was to study the correlation between the temporal organization of quiet sleep (QS) and maturation in premature infants without severe complications during their neonatal hospitalization.

**Methods:** Percentage of time spent in QS and average duration of time intervals (ADI) spent in QS were analyzed from a cohort of newborns with no severe complications included in the Digi-NewB prospective, multicentric, observational study in 2017–19. Three groups were analyzed according to GA: Group 1 (27–30 weeks), Group 2 (33–37 weeks), Group 3 (>39 weeks). Two 8-h video recordings were acquired in groups 1 and 2: after birth (T1) and before discharge from hospital (T2). The annotation of the QS phases was performed by analyzing video recordings together with heart rate and respiratory traces thanks to a dedicated software tool of visualization and annotation of multimodal long-time recordings, with a double expert reading. Results are expressed as median (interquartile range, IQR). Correlations were analyzed using a linear mixed model.

**Results:** Five newborns were studied in each group (160 h of recording). Median time spent in QS increased from 13.0% [IQR: 13–20] to 28.8% [IQR: 27–30] and from 17.0% [IQR: 15–21] to 29.6% [IQR: 29.5–31.5] in Group 1 and 2, respectively. Median ADI increased from 54 [IQR: 53–54] to 288 s [IQR: 279–428] and from 90 [IQR: 84–96] to 258 s [IQR: 168–312] in Group 1 and 2. Both groups reach values similar to that of group 3, respectively 28.2% [IQR: 24.5–31.3] and 270 s [IQR: 210–402]. The correlation between PMA and time spent in QS or ADI were, respectively 0.73 (*p* < 10^−4^) and 0.46 (*p* = 0.06). Multilinear analysis using temporal organization of QS gave an accurate estimate of PMA (*r*^2^ = 0.87, *p* < 0.001).

**Conclusion:** The temporal organization of QS is correlated with PMA in newborns without severe complication. An automated standardized continuous behavioral quantification of QS could be interesting to monitor during the hospitalization stay in neonatal units.

## Introduction

Fifteen million babies are born preterm every year around the world and the overall rate of preterm live births is 11%. In Europe, the median preterm birth rate is 7.3% (1). Although the brain structures are formed, premature birth interrupts the normal maturation process of the central nervous system. Among the axes of preterm newborn maturation, sleep is a major contributor to the development of neural pathways in the neonatal brain (2).

The two main sleep stages for the newborn are Active Sleep (AS) and Quiet Sleep (QS). Both are involved in development, maturation, and connectivity within neural networks in the brain with impacts on memorization, consolidation of learning capacities and plasticity (3, 4). Despite the importance of sleep on neuro-development little is known about development and impact of sleep alterations in very preterm newborns. The standard environment of the hospitalized newborns is complex and differs significantly from the *in utero* environment. In neonatal units, many interventions are required and the preterm infants are exposed to many alarms that can disturb the quality and quantity of sleep. Sleep deprivation is known to have a negative impact on health and neuro-cognitive functions and to increase the risks of cardio-respiratory events occurring during the hospitalization (4). It is also known that infants born preterm have a high risk to have altered sleep patterns after the neonatal period with for example a decrease of wake-up calls in QS which could be a predisposing factor for sudden infant death syndrome (5).

The studies performed in preterm newborns are mainly based on the behavioral classification of the stages of alertness described by Prechtl (6) and Brazelton (7) or on studies using polysomnography or actigraphy. Well-defined periods of AS and QS have been detected as early as 27 weeks of gestational age (GA) (8) and studies show an increase in the proportion of time spent in QS and wakefulness with maturational age with different amplitudes of changes depending on the methods used, the inclusion criteria and the study date (9–11). In clinical practice the measurements of the sleep phases remain challenging and the impact of the studies are limited by the heterogeneity of sleep assessment. Polysomnography measurements are carried out over a limited period of time, in a highly standardized environment with many measurements and sensors that limit its usefulness to follow the maturation. The other alternative includes detailed clinical observation as in neonatal intervention programs, the use of actigraphy or amplitude-integrated EEG and the recent introduction of new methods for automatic classification of neonatal sleep states based on EEG (12–14). Some interventions have been proposed for sleep promotion but their evaluation is limited by the absence of reliable longitudinal monitoring (15–17).

We hypothesized that the temporal organization of QS could be a reliable, reproducible, easily and non-invasively accessible marker of sleep maturation for preterm infants hospitalized in neonatal units. As a first step to this approach, we set this study to annotate QS periods from recordings of video and vital physiological signals (i.e., ECG and respiration) during maturation of preterm infants. The objective was to demonstrate the correlation between postmenstrual age (PMA) and the temporal organization of the QS using a non-invasive approach. We wanted to avoid additional electrodes or adhesive patches and cables attached to the premature newborn to avoid skin lesions and induce sleep disorders (2). A dedicated software was built to provide multimodal visualization and an annotation tool.

In this paper, we propose a 2-fold contribution with a study of the temporal organization of QS of preterm newborns, and the description of a new software tool for video and signal visualization and sleep stage annotation. The temporal organization of QS is described according to the percentage of time spent in QS and the average duration of intervals (ADI) spent in QS which have been studied as a function of GA and PMA. The development of a configurable software for multimodal signal visualization and annotation named ViSiAnnoT (Video Signal Annotation Tool) allows to visualize as many signals and videos as wanted and handles modalities that are not initially necessarily synchronized with each other. It also provides a multi-label annotation tool of temporal events.

## Materials and Methods

### Ethical

The data used in this study was part of the database of the Digi-NewB observational cohort (NCT02863978, Horizon 2020 European project GA-689260). This cohort prospectively included newborn infants hospitalized in the neonatal units of six University hospitals in the western region of France (University Hospitals of Rennes, Angers, Nantes, Brest, Poitiers, and Tours) in 2017–2019. The collection of the data was carried out after approval by the ethics committee (CPP Ouest IV 34/16), national agency for the safety of medicines and health products (Authorization number: 2016062400181) and informed parental consent. Digi-NewB project aimed at improving clinical intervention in neonatal units by developing a new generation of non-invasive perinatal health monitoring based on multi-parametric digital representation of clinically relevant functions. The objective was to assist clinicians in their decision-making of cardio-respiratory and neurobehavioral maturation.

### Population

The newborns included in this study were part of the Digi-newB cohort which allowed inclusion of hospitalized newborn infants aged of <47 weeks PMA. They were chosen to highlight differences in maturation according to GA and PMA. GA was defined as the time elapsed between the 1st day of the last menstrual period and the day of delivery; PMA was defined as GA plus postnatal age (18).

Newborns presented with identified neonatal complications, maternal cardiac or neurological treatment and maternal drug abused were not included in the study. Complications that led to exclusion were: intraventricular hemorrhage, white matter lesions, abnormal brain imaging at term PMA, bronchopulmonary dysplasia defined as ventilatory support or oxygen requirement at 36 weeks PMA, enterocolitis and duration of anti-infectious therapy of more than 5 days during hospital stay and congenital malformation. The newborns were also excluded if the quality of video or signal recordings was insufficient (i.e., lack of visibility of the newborn over the entire recording hampering descriptive analysis or artefacted ECG and respiratory traces). The included newborns were divided into three groups according to GA with five newborns in each group. Group 1 consisted in very preterm newborns born with GA between 27 and 29 weeks + 6 days, group 2 consisted in late preterm newborns with GA between 33 and 36 weeks + 6 days, group 3 consisted in healthy full-term newborns with GA between 39 and 40 weeks. For all newborns, a recording was acquired during the 1st week after birth (recording time one: T1). For newborns of groups 1 and 2, a second recording was acquired before discharge (recording time two: T2). QS phases were characterized from video recordings, heart rate and respiratory traces through a dedicated software (ViSiAnnoT), with a double expert reading.

### Recordings

All newborns were studied on continuous 8-h periods recorded between 10-p.m. and 6-a.m. These periods were chosen because it is known that the initial ultradian periodicity is around 4 h with sleep-wake cycles around 1 h during the 1st days of life (19, 20) and because sleep is less interrupted at night than during the day in neonatal units (21). Continuous infrared video (two cameras), ECG and respiration signals were together acquired. The ECG was obtained with a sampling rate of 500 Hz in order to obtain time series of the cardiac cycle lengths (RR intervals) on a beat-to-beat basis. The RR intervals were detected using a modified version of the Pan and Tompkins algorithm, with filter coefficients specifically adapted for newborns (22). For this study the analysis of heart rate variability was made by visual analysis of the beat to beat tachogram. During the preliminary phases of this study in the framework of the Digi-NewB project we were able to evaluate the performance of the use of infrared cameras for motion detection and sleep evaluation. This was done by comparing the evaluations made by Nidcap certified nurses and the observation of videos as well as by comparing the motion curves obtained in automatic detection and the observation of videos (23). We concluded from these studies that it was possible to have a reliable evaluation of movement and sleep stages if two viewing angles and thus two cameras were used. We then established the constraints to adapt the use of two cameras in the different environments observed in neonatology (open or closed incubator, cradle.). The set-up of the cameras required no interaction with the newborn and was adapted to neonatal environment not to disturb the medical and paramedical team.

### Video and Signal Annotation Tool

The use of a software tool for multimodal visualization and annotation eases the process of manual scoring of QS periods. For this study, we have built a software tool which allow to display video and physiological signals in a synchronized way, while integrating a tool for annotating temporal events.

There are only a very few software tools that allow a multimodal visualization, such as RTGraph (24) (physiological signals), CAPTIV (25) (video, motion curve, audio, and hypnogram) or Natus NeuroWorks (EEG, video) (26). Nevertheless, they do not integrate an annotation tool. We can find some annotation tools in the domain of affect computing, such as FEELTRACE (27), CARMA (28), or ANNEMO (29). The drawback is that they are designed to visualize only audio-visual recordings, so we cannot combine it with the visualization of physiological signals. Regarding software distribution, RTGraph (24) is the only one being open source. Open source distribution has many advantages such as: being available to everyone, not depending on a third party software requiring a license and allowing collaborative work for improving the software and adding features. To the best of our knowledge, there is no software tool that meet all of our requirements.

So, we developed a software tool for multimodal signal visualization and annotation: Video and Signal Annotation Tool (ViSiAnnoT). It has been used in this study in order to score QS periods. The main advantage of ViSiAnnoT is to provide an annotation tool that eases the scoring process in a research study.

**Figure 1:**
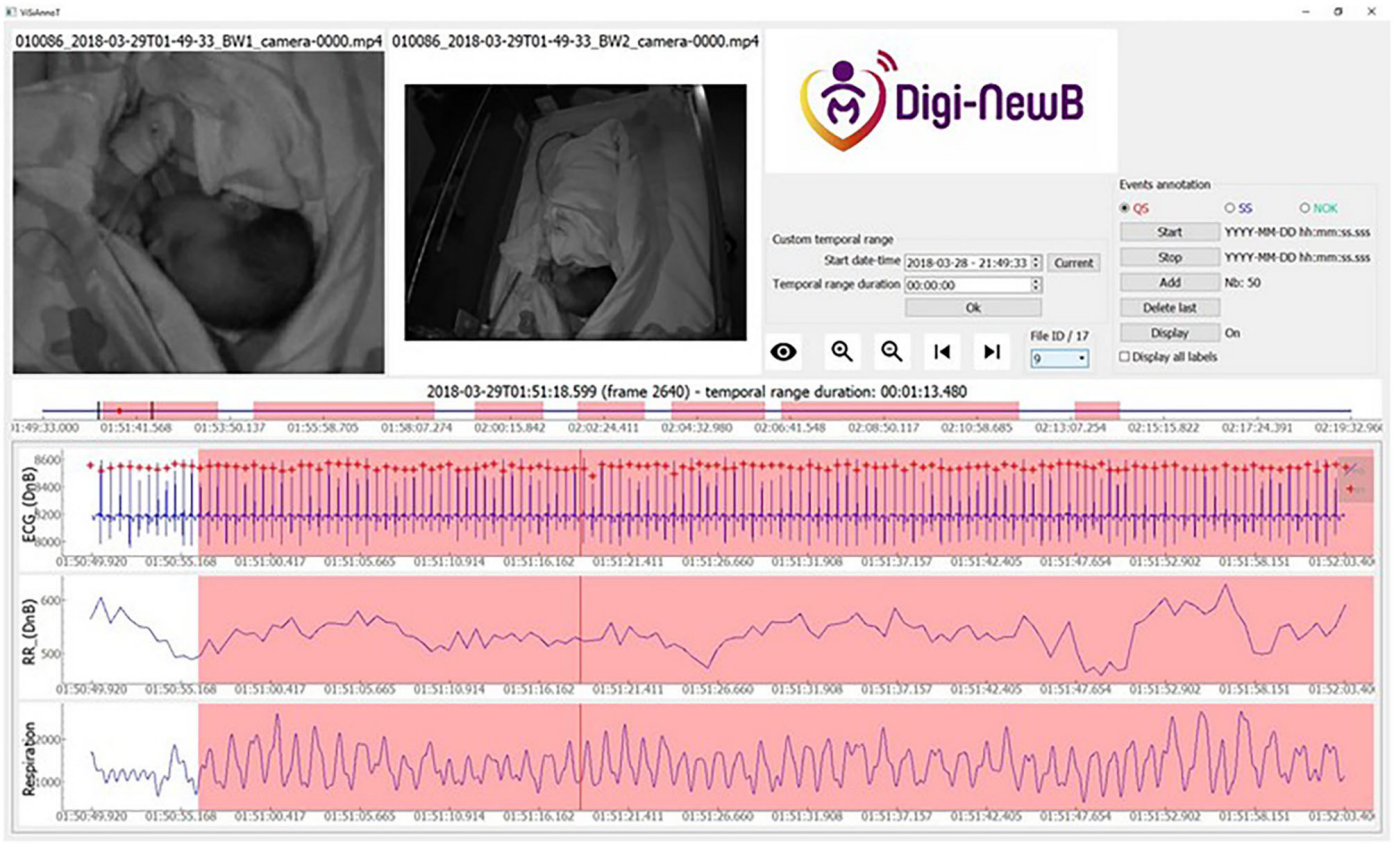
Screenshot of ViSiAnnoT software displaying two videos and three signal plots, for a preterm infant at 35+5 days PMA. We used ViSiAnnoT to display two synchronized videos and three signals (ECG, RR time series, and respiration). In the progress bar (between the videos and the signal plots) there are seven annotated quiet sleep (QS) intervals in the 30-min file. Annotated quiet sleep interval is highlighted in red.

The first main feature was to provide a combined video and signal multimodal visualizer. Several synchronized videos can be displayed, and it is possible to display together several signals regularly sampled or not on multiple or single plots. An example of ViSiAnnoT visualization of non-regularly sampled signal is shown in Figure 1 which presents the visualization of RR intervals time series (cardiac cycle lengths) extracted from the ECG together with video and other plots. The supported formats for video files are those supported by OpenCV (30). The supported formats for signal files are txt, mat (Matlab) and h5. The software is designed to work off-line, meaning that the signals must be extracted beforehand and cannot be retrieved directly from the acquisition system.

The second main feature of ViSiAnnoT is the management of long recordings. In the context of this study, each recording was split into files of 30 min length, so we integrated a user interaction that allows to navigate easily from one file to another with buttons “next” and “previous.” Moreover, there is a combo box for directly selecting a specific file in the recording and the user can specify if the different signal modalities are synchronized with another or not.

The third main feature is the multi-label annotation tool, which allows to annotate temporal intervals. This tool was built to characterize frequency of occurrence and duration of specific events, as it is the case in this study. It automatically creates a file for each label, where the annotations are written. The annotations can be displayed on top of signal plots with colored intervals, each color corresponding to a label.

Even though the software has been developed in the context of this study, it is designed to be highly configurable regarding the number of signals to display, the plot style, the annotation labels and the general layout. Thus, it can easily adapt to a wide range of applications where it is needed to annotate temporal events. ViSiAnnoT mainly relies on PyQt5 package (Python binding for Qt) (31) and PyQtGraph package (32). For someone with background knowledge in these packages and in object-oriented programming, it should be quite easy to add new features. So, we chose to distribute ViSiAnnoT as an open-source software, so that the user can adapt it to his particular needs. Compared to commercial system, ViSiAnnoT most likely has poorer performances and provides less features, but it is easy to use, freely available and is highly configurable. The software is currently in the beta phase.

### Sleep Scoring

The scoring of QS was primarily based on Brazelton neonatal Behavioral assessment Scale (7). Some specific rules for annotation were established on a learning base of eight recordings by two experts. Classification has been established over periods of at least 20 s epochs. QS was defined as a period without spontaneous motor activity, when eyes are closed and without visible eye movement. The face should not be expressive, but relaxed, except for rare short isolated periodic movements of sucking. It is also required to have a regular heart rate and respiratory rate. Although there is no established definition of the regularity of cardiac and respiratory frequencies, we used as reference the proposal of Anders et al. (33). A relatively regular respiration is a period when the rate varies from <20 cycles per minute. We also took into account the regularity of respiratory amplitude and cardiac tachogram. We considered the periodic changes in breathing pattern and heart rate seen during periodic breathing to be regular breathing.

Startles and sighs are physiologically present during QS in preterm and term newborns. Startle is characterized by a spontaneous or reflecting motoric symptom like brisk, short lasting and generalized contraction of limb and trunk muscles influencing considerably the cardio-respirogram of neonates (34). A startle usually lasts <5 s but it can be followed by a relaxation phase of a few seconds (35). Sighs are deep breaths that are common in newborns and are often followed by apnea and hypoventilation (36). Since a QS phase is defined by a period with no motor activity which lasts at least 20 s, startles and sighs should not be considered as a standard motor activity in order not to incorrectly split QS phases. So, they are considered as part of QS phases during the annotation. In the context of developing a video-based automatic method for QS detection, it is critical to be able to characterize startle and sighs, which is why we annotated them in parallel with the QS phases.

**Figure 2:**
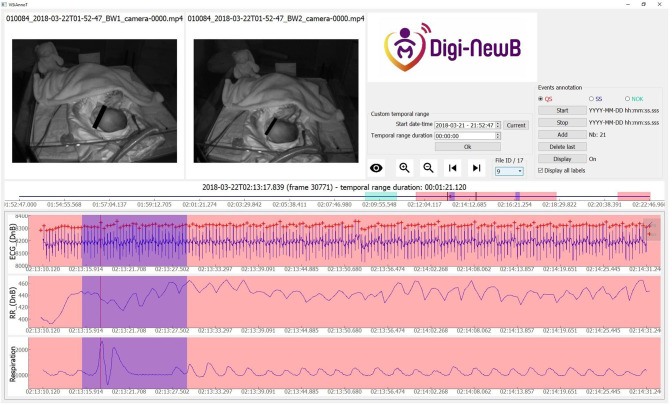
Screenshot of ViSiAnnoT software illustrating a startle in a newborn at 37+1 days PMA. There is a short interval of motion (visible in videos) with a modification of respiratory and ECG cycle for startle. The annotated startle intervals are highlighted in blue.

**Figure 3:**
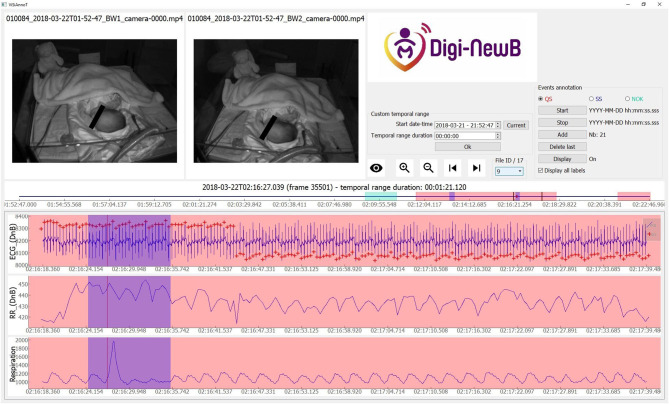
Screenshot of ViSiAnnoT software illustrating a sigh in a newborn at 37+1 days PMA. There is a short interval of motion (visible in videos) with a modification of respiratory and ECG cycle for sigh. The annotated sigh intervals are highlighted in blue.

We used ViSiAnnoT to display two synchronized videos and three signals (ECG with TQRS detection, RR series and respiration. Three labels were used for annotation: QS (quiet sleep) (Figure 1), SS (startles and sighs) (Figures 2, 3), and NOK (“*not OK:”* periods that are not suitable for analysis). The NOK label was used in three situations: newborn outside the camera view, missing respiratory or ECG signals, adult present in the field of view of the cameras. All the QS scoring were validated by a double reading involving the two experts. The QS was evaluated independently and then the two experts compared all their quotations in specific analysis sessions.

### Statistical Analysis

Statistical analysis was performed using Statistica 13.2 (StatSoft Inc.) and SAS 9.4 (SAS Institute). All variables were tested for normal distribution using the Shapiro-Wilk normality test. Variables were analyzed by paired or unpaired Student's *t*-test, or Wilcoxon w-test and Mann-Whitney u-test as appropriate. We first tested if characteristics of QS (i.e., ADI and % time spent in QS) were correlated with maturation measured by PMA. The % of QS was calculated on the whole usable recording after removing the NOK periods from the denominator. Correlations between data were calculated considering repeated data. Then we tried to get an index which could be clinically useful to estimate sleep maturation in terms of post-menstrual equivalent age (expressed in estimated weeks of PMA) using ADI, % of time spent in QS and GA as independent variables. This was tested using multiple-linear regression analysis with and without inclusion of GA. The variables were chosen according to an a priori hypothesis without any prior selection process. In the model “gestational age,” “ADI,” and “% of time spent in QS” are the fixed effects and the patient the random effect. A linear mixed model was used to take into account the repeated measurements in groups 1 and 2. The model used a restricted maximum likelihood method in which the variance structure is variance components. The formula used to calculate R-squared was as follows: R-squared = 1- VAR (residual)/VAR (total) and specifically required a type 3 estimation method.

The significance level was set at 0.05. The results are presented as mean (Standard Deviation) or as median (interquartile range: IQR).

## Results

### Newborn and Maternal Characteristics

Infant and maternal characteristics are presented in Table 1. Fifteen newborn infants were studied with two recordings for each newborn included in group 1 and 2 and one recording for full-term newborns (group 3). All the preterm newborns (group 1 and 2) were in an incubator breathing spontaneously at T1. All preterm infants from group 1 and two in group 2 were assisted by continuous positive airway pressure at T1. All the newborns were in cradles breathing spontaneously without ventilator support during the other recordings.

**Table 1:** Infant and maternal characteristics.

	**Preterm infants**		**Term infants**
	Group 1 (27–30) wk	Group 2 (33–37) wk	Group 3 (>39) wk
	*n* = 5	*n* = 5	*n* = 5
**Gestational Age at Birth**, wk			
Median, IQR [Q1–Q3]	28+2 [27+6 – 28+5]	33+5 [33+3 – 35+4]	40 [39+4 – 40+3]
**Delivery Mode**			
Cesarean	3	1	3
Vaginal	2	4	2
**Antenatal Corticoids**	5	3	-
**Antenatal Magnesium sulfate**	3	0	-
**Sex**			
Female	2	1	2
Male	3	4	3
**Apgar Score at 1 min**,			
Median, IQR [Q1–Q3]	8 [2.5–8.5]	8 [5.5–9.5]	8 [2–9]
**Apgar Score at 5 min**,			
Median, IQR [Q1–Q3]	9 [8–9.5]	10 [6–10]	8 [4.5–10]
**Caffeine treatment**	4	2	0
**Birth Weight** (g)			
Median, IQR [Q1–Q3]	930 [925–1,275]	2,270 [2,260–2,425]	2,850 [2,550–3,060]

*All data are presented as median and IQR, interquartile range; wk, week; g, gram*.

### Analysis of Quiet Sleep Duration According to Maturation

All of the pre-selected 8-h periods were visually analyzed. The time spent by the observers to score QS was ~450 h.

**Figure 4:**
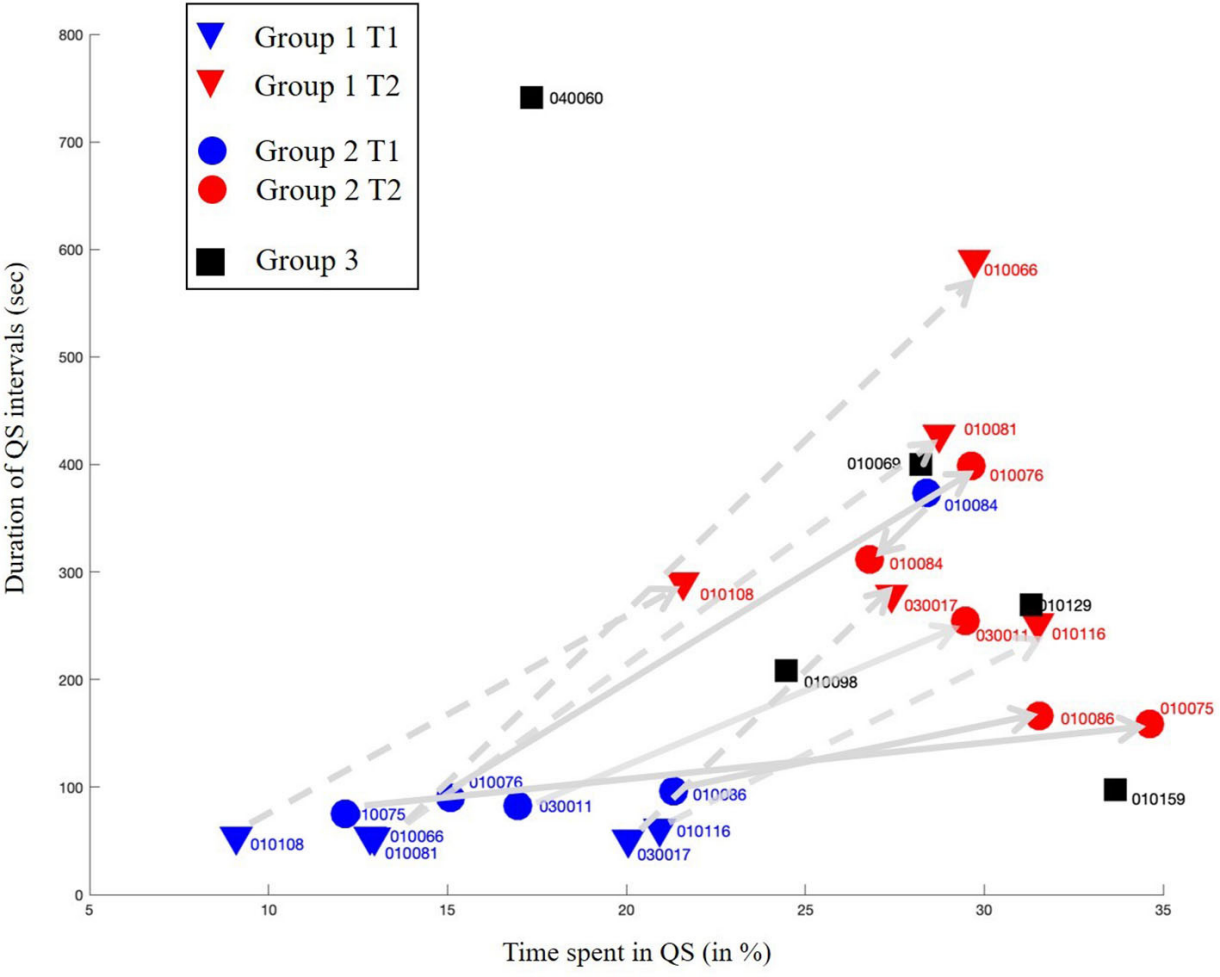
ADI of quiet sleep intervals according to the percentage of time spent in quiet sleep (QS). Arrows show the evolution of the values between T1 and T2 for each newborn of group 1 (dashed line) and group 2 (solid line). There is no increase in QS percentage for the preterm infant N°010084, this could be explained by a short period between the two recordings on his gestational age which represents a late preterm of group 2.

The individual results concerning percentage of time spent in QS and ADI spent in QS are presented in Table 2 and in Figure 4. Time spent in QS and ADI were not significantly correlated (*r* = 0.38, *p* = 0.06).

**Table 2:** Average duration of quiet sleep intervals and percentage of time spent in quiet sleep.

**Group**	**Patient ID**	**QS episodes /recording**		**Post-menstrual age (weeks)**		**ADI spent in QS (seconds)**		**Time spent in QS (%)**	
		**T1**	**T2**	**T1**	**T2**	**T1**	**T2**	**T1**	**T2**
1	1108	55	13	28.6	36.9	54	288	9.1	21.6
	1116	58	17	28.6	38.1	60	252	20.9	31.5
	1066	32	15	28.7	38.1	54	588	13	29.7
	3017	69	20	28.7	38.0	54	282	20.1	27.4
	1081	56	30	29.7	37.3	54	426	12.8	28.8
2	1075	48	46	34.1	37.1	78	156	12.1	34.6
	1076	21	24	33.6	36.6	90	402	15.1	29.6
	3011	50	48	33.9	36.9	84	258	17	29.5
	1086	42	22	35.7	37.7	96	168	21.3	31.5
	1084	45	17	37.1	38.1	372	312	28.4	26.8
3	1159	9		39.9		96		33.7	
	1098	30		39.9		210		24.5	
	4060	21		40.1		744		17.4	
	1069	7		40.7		402		28.2	
	1129	69		41.4		270		31.3	

Group 1: very preterm newborns born with GA between 27 and 29 weeks + 6 days. Group 2: late preterm newborns with GA between 33 and 36 weeks + 6 days. Group 3: healthy full-term newborns with GA between 39 and 40 weeks. Two values are given for groups 1 and 2: at recording Time 1 (T1) and Time 2 (T2).

*ADI, average duration of intervals; QS, quiet sleep*.

**Figure 5:**
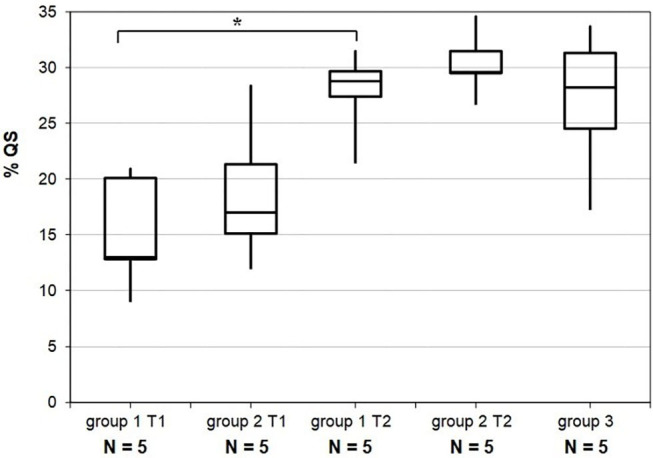
Results for the percentage of time spent in quiet sleep. Distributions for each group and according to recording time T1 and T2. Horizontal lines grouping a pair of boxes denote statistically significant differences (*p < 0.05) in a Mann-Whitney *u*-test. % QS, percentage of quiet sleep; GA, gestational age; PMA, post-menstrual age; T1, recording time 1; T2, recording time 2.

Percentage of time spent in QS increased between T1 and T2 for all preterm newborns except one. This increase was significant (*w*-test, *p* < 0.01) for the whole preterm population studied. Median time spent in QS increased from 13.0% [IQR: 13–20] to 28.8% [IQR: 27–30] in Group 1 (w test, *p* < 0.05) and from 17.0% [IQR: 15–21] to 29.6% [IQR: 29.5–31.5] in Group 2 (*w*-test, *p* = 0.08). Median time spent in QS in group 3 was 28.2% [IQR: 24.5–31.3] and not significantly different from T2 in groups 1 and 2 (Figure 5). The percentage of time spent in QS was correlated with PMA (*r* = 0.73, *p* < 0.0001).

**Figure 6:**
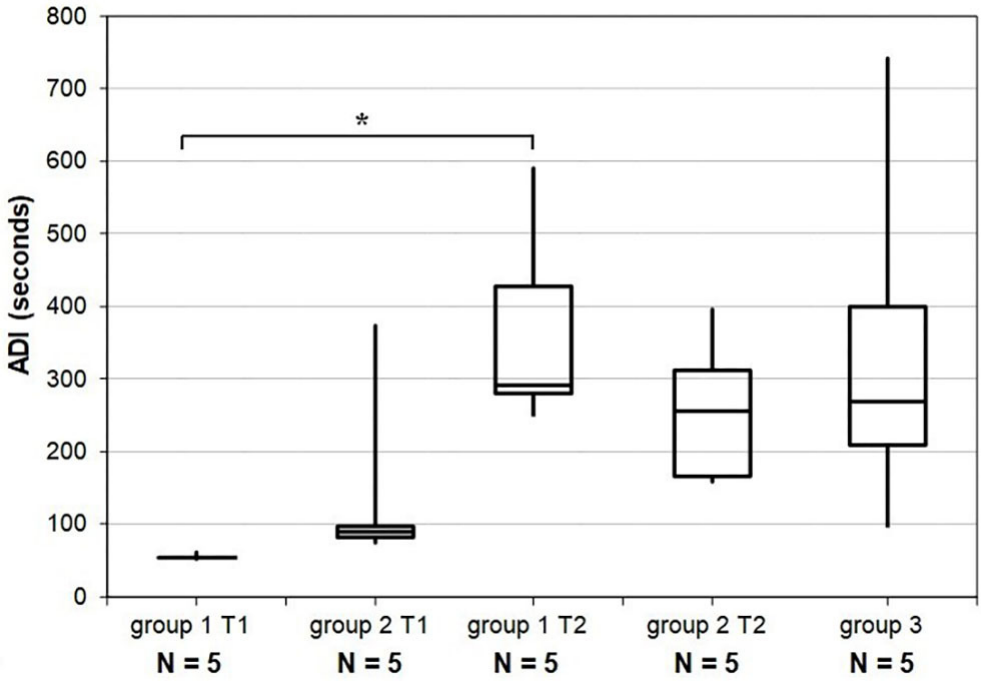
Results for the average duration of intervals spent in quiet sleep. Distributions for each group and according to recording time T1 and T2. Horizontal lines grouping a pair of boxes denote statistically significant differences (*p < 0.05) in a Mann-Whitney *u*-test. ADI T1, average duration of intervals at recording time 1; ADI T2, average duration of intervals at recording time 2; GA, gestational age; PMA, post-menstrual age.

ADI spent in QS, expressed in seconds, was lower at T1 in group 1 than in group 2 (*u*-test, *p* < 0.05) and increased between T1 and T2 for all preterm newborns except one. This increase was significant (*p* < 0.01) for the whole preterm population studied. Median ADI spent in QS increased from 54 s [IQR: 53–54] to 288 s [IQR: 279–428] in Group 1 (*w*-test, *p* < 0.05) and from 90 s [IQR: 84–96] to 258 s [IQR: 168–312] in Group 2 (*w*-test, *p* = 0.08). Median ADI spent in QS in group 3 was 270 s [IQR: 210–402] and not significantly different from T2 in groups 1 and 2 (Figure 6). The ADI spent in QS was not significantly correlated with PMA (*r* = 0.46, *p* = 0.06). The increase in the ADI with age was mainly due to the decrease in short QS episodes before term. In group 1 and 2 at T1 69% (IQR: 68–77), and 48% (IQR: 40–49) of the ADI were between 20 s and 1 min. At term equivalent age these percentages were of 10% (IQR: 8–13), 17% (IQR: 14–22), and 17% (IQR: 0–37) in groups 1, 2, and 3, respectively.

### Estimation of PMA

**Figure 7:**
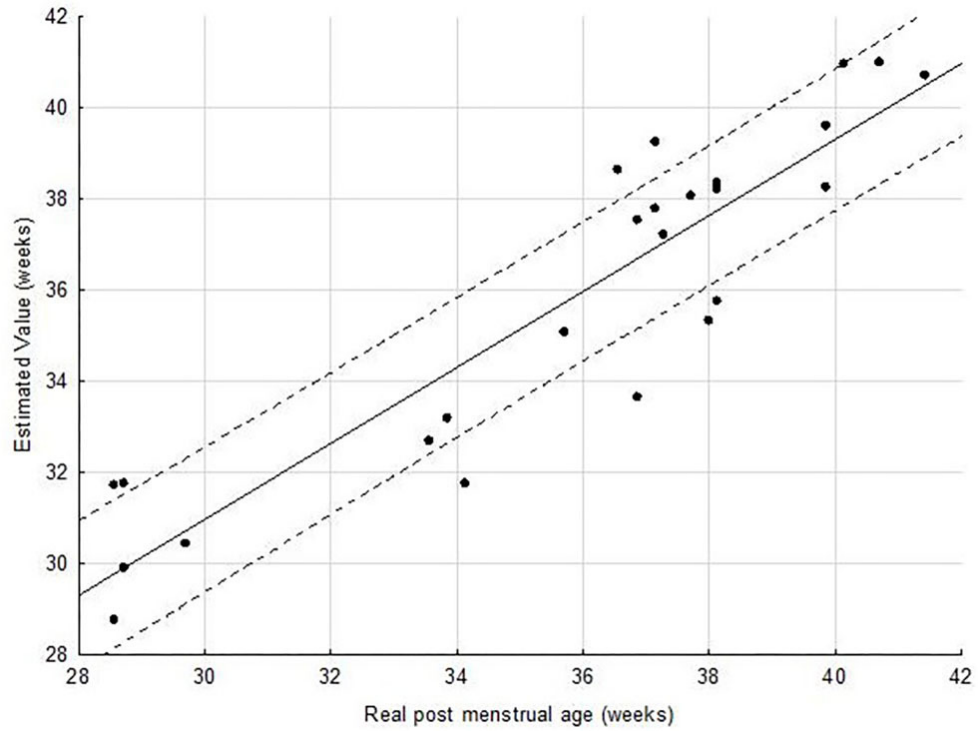
Scatter plot of estimated brain-age against PMA. The solid line indicates the regression line and the dashed line indicates the 0.95 confidence interval of the regression line. The mean standard error of the estimate was 1.76 weeks.

The correlation coefficient was *r*^2^ = 0.71 (*p* < 0.001) with the primary mixed model of multiple linear regression analyses for estimation of PMA using percentage of time spent in QS and ADI spent in QS as independent variables (Figure 7 and Table 3).

**Table 3:** Results of multiple linear regression analyses estimated brain-age from quiet sleep characteristics against post-menstrual age (PMA = gestational age + postnatal age).

***N* = 25**	Béta value (Std^**^ Error)	Co-efficient (Std^**^ Error)	***T* ratio**	**probability**
Constant		26.5 (1.6)	5.74	0.00001
**Independent Variables**				
Duration of QS^*^ intervals	0.43 (0.13)	0.58 (0.18)	3.25	0.004
% of QS^*^	0.55 (0.13)	0.30 (0.07)	4.18	0.0004
*R*^2^		0.67	(0.83^§^)	
*R*^2^ linear mixed model		0.71	(0.87^§^)	
*F* ratio		22.5	(35.3^§^)	
Std^**^ error of estimation		2.4	(1.8^§^)	
*P*-value		<10^−7^	(<10^−7§)^	

* QS, quiet sleep.

** Std, standard.

§ *value of R^2^ if GA is added in the model. The results of R^2^ is presented for the primary model of multiple linear regression analyses for estimation of PMA using percentage of time spent in QS and ADI spent in QS as independent variables. The R^2^ values presented between brackets corresponds to the estimation of PMA in adding GA, which was correlated with PMA (r = 0.66), to the primary model*.

As expected, this correlation significantly increased (*p* < 10-4) to a value of *r*^2^ = 0.87 (*p* < 0.001) when estimation was made using a second model in adding GA which was correlated with PMA (*r* = 0.66) to the primary model.

## Discussion

In this prospective, multicenter, observational, cohort pilot study, involving a small number of preterm infants selected to be representative of the optimal expected maturation trajectory in preterm infants we have shown that percentage of time spent in QS and ADI spent in QS increased as a function of PMA. The temporal organization of QS appears correlated with PMA and depends on postnatal maturation in newborns without severe complication. QS, as assessed in this study, could therefore potentially be used as a clinical tool for milestone development. This result was obtained thanks to the ViSiAnnoT software tool for signal visualization and annotation.

The American Academy of Sleep Medicine have recently proposed rules for sleep scoring in full-term infants from birth to 2 months of age based on eye status, chin EMG, behavior and EEG but the criteria used require invasive procedures that are not applicable for monitoring of sleep maturation in clinical practice (37). Some authors such as Prechtl, Anders or Brazelton (6, 7, 33) have proposed non-invasive behavioral evaluation to scale sleep but the proposed criteria have not been uniformly applied across the neonatal studies. This could be explained by the lack of precision in the proposed definitions and by the fact that there is no specific recommendation for measurement of sleep stages applicable to the preterm infant. The method used to qualify QS phases has an impact on the reported temporal organization of QS. In our study, we applied a standardized method to qualify QS all along the traces on pre-established criteria that were selected from the literature with precise definition refined in an initial learning phase. We used criteria for definitions of regularity of respiration, startles and sighs, that are not usually described in the previous publications studying sleep in preterm infants. One limit to our study could have been absence of concordance study between the experts but the QS annotation has been done following a consensus method to get a unique judgement criteria. The time interval used to qualify sleep stages also varies, depending on the study, from 20 s to 1 min (35, 38). We have chosen a minimum period of 20 s for the annotation of a QS phase. This choice of a short period has an impact on both ADI spent in QS and percentage of time spent in QS, for example it allows detection of a significant number of short QS period for very preterm infants. With the definitions chosen in the current study, we observed a high correlation between QS characteristics (i.e., percentage of time spent in QS and ADI) and PMA which suggests a clinical relevance for estimation of sleep maturation in preterm infants. Most of the publications on sleep in preterm infants are EEG-based (39, 40). In literature, currently used behavioral scales have shown to have a low concordance with sleep based on EEG parameters (40). All together, these observations suggest that it would be clinically useful and relevant to set a behavioral-based automated non-invasive bedside tool for QS evaluation.

In this longitudinal study, we presented a behavioral measurement of the observed changes in temporal organization of QS during the neonatal period. Most of the previous studies concerning the temporal organization of QS had been carried out by non-longitudinal EEG evaluations. The results of the current study are in line with the reported results using invasive methods. In the 80's, studies converged to say that there would be a significant increase in the percentage of QS as a function of PMA. According to Stefanski, QS rose from 18.9% before 36 weeks of PMA to 30.4% after 36 weeks of PMA (11). According to Curzi-Dascalova, QS rose from 27% at 31–34 weeks PMA to 33% at 35–36 weeks PMA (*p* < 0.05) (9). In our study the time spent in QS was around 13% for very preterm infants before 30 weeks PMA, around 17% before 37 weeks PMA, and around 29% at term-equivalent age without significant differences related to GA. These results are close to the results of Hoppenbrouwers et al. obtained by using polysomnography who reported percentage of time spent in QS of 18% for 34–37 weeks PMA for and of 30% at term equivalent age (41). These results are also close to the published results for healthy term infants. Korotchikova et al. using video-EEG including behavioral and EEG aspects reported a mean percentage (SD) of QS of 38.6 (12.5)% (42).

The ADI spent in QS is not well-documented in the literature concerning preterm infants in the neonatal period. Using 30 sec-epoch annotations from polysomnography obtained from 193 preterm infants, Hoppenbrouwers found that the mean duration of all QS episodes showed a significant trend with a rise from 6 min at 34–37 weeks PMA to 11 min at 50–53 weeks PMA (41). Our longitudinal study gave new information about the changes in ADI in hospitalized preterm infants. We have chosen a minimum period of 20 s for the annotation of a QS phase as QS periods are known to be short and sometime difficult to distinguish from intermediate sleep during the early stages of hospitalization of very preterm infants. In the literature, the time interval used to qualify sleep stages in preterm infants varies from 10 s to 1 min (35, 38, 41, 43, 44). When looking at the periods of QS with duration between 20 s and 1 min we observed that the choice of a short period for annotation has an important impact on both ADI and percentage of time spent in QS. This, together with the difficulties to separate intermediate sleep from QS in some cases, has to be taken into account when comparing the studies involving preterm infants and could at least in part explain some discrepancies between the published studies particularly in the early stages of hospitalization of very preterm infants.

We confirmed that maturation of preterm infants is significantly associated with an increase in ADI that begins in the early time of hospitalization. This increase was mainly due to the decrease in short QS episodes before term. We also observed that, in preterm infants presenting an uncomplicated course during hospitalization, ADI as well as percentage of time spent in QS observed at term equivalent age did not differ depending on GA. This result differs from the study of Scher et al. in the 1990's, which reported a significantly higher rate of QS measured from EEG in preterm infants at the equivalent age at term than in term infants (34 vs. 28%, *p* = 0.02) (45).

Many factors are known to alter sleep in the neonatal period. We took care to limit the confounding factors that could interfere with the relation between QS temporal organization and maturation but some of them remained. In our study, all the very preterm were exposed to antenatal steroids and 60% to antenatal magnesium sulfate (MgSO_4_), and 60% of the late preterm infants were also exposed to antenatal steroids. The effects of these antenatal treatments have been studied in a longitudinal study including 134 preterm infants from 35 to 45 weeks PMA (46). In this study, prenatal exposure to MgSO_4_ was associated with fewer state changes per hour and an increase in respiration regularity but without other alteration in variables related to QS organization. Caffeine is known to improve survival free of neurodevelopmental disability at 18 to 21 months' PMA (47) and is therefore largely used to treat apnea of prematurity (48), but little is known about the effects of chronic caffeine treatment on sleep of preterm infants. In our study, 80% of very preterm infants and 40% of late preterm infants were treated by caffeine. Hayes et al. have evaluated the impact of caffeine on sleep of preterm infants at 33–34 weeks PMA in a small sized study (49). Their results using behavioral actigraphic and videographic 5-h recordings in 10 very preterm infants suggest that caffeine treatment could be associated with a decrease in both arousal rates and wakefulness (49). Another limit of our study is that some other variables such as sex, ventilatory support, maternal smoking, small for GA or time from birth could have influence sleep architecture but are not accessible from our study (50–52). The number of newborns included in our study was relatively small, but they were strictly selected to be representative of the optimal expected maturation trajectory in preterm infants. We only involved newborns with an uncomplicated course of hospitalization. Moreover, we have used a standardized analysis of long periods of recordings obtained in the usual environment inside neonatal units which required a high amount of time for annotation. With this approach we observed a high correlation between QS organization and PMA with and without inclusion of GA as an independent variable arguing for a clinical relevance of the method of annotation used. It is to note that in a statistical perspective the inclusion of GA which is correlated with the PMA in the predictor artificially increase the correlation but we think that the inclusion of this variable in the model remains useful in a clinical perspective. The variability of the time interval between T1 and T2 may have slightly affected the concept of repeated measurements. However, we find these preliminary results informative in view to develop an automated detection method. Our proposal to evaluate behavioral approach with regard to correlation with PMA is not unique, it has been recently used in two studies using EEG (53, 54). The observed correlation were also high in those studies and quite similar than in our study. In the study of Pillay et al. (53) a 10–20 electrode EEG system was used to quantify EEG maturation of QS evaluated on 30 s epoch with a Median recording duration of 4 h 23 min in 21 preterm infants using a data-driven approach based on EEG features which didn't take into account percentage of time spent in QS or ADI (69 recordings). The observed *r*^2^ value of correlation between the estimated brain age and the PMA was of 0.85–0.88. In the study of Han et al. (54), estimated maturity of sleep state was also correlated with PMA both on conventional electroencephalography, cEEG (*r*^2^ = 0,86) and amplitude-integrated electroencephalograph, aEEG (*r*^2^ = 0.72) in 51 3–4 h recordings. The definition of QS is less and less easy to define as term decreased under 30 weeks gestation, especially in terms of duration and transition between the sleep stages. Furthermore, the way to evaluate QS during these periods could be challenged. It is one of the reasons why we have chosen to analyze sleep by periods of 20 s with very detailed observational criteria. Despite this, we observed that PMA estimation is less accurate before 30 weeks of observed PMA and that evaluation of QS is probably more accurate and usable after 30 weeks. We think therefore that the annotated database built in this study can be used as a learning base to set an automated multiparametric detection of QS phases based on video, heart rate and respiratory rate analyses. Such a method could be useful for a non-invasive continuous evaluation of normal and abnormal sleep maturation in neonatal units. The behavioral approach requires a trained observer and is known to be subjective, expert-dependent and time consuming. This observational approach is difficult for non-expert in the field with limited data concerning accuracy of the evaluation. Overall agreement among assessors and agreement between behavioral observation and polygraphy appear to be low, at least for untrained staff and especially as the newborn is more premature and registration is shorter (55). It seems therefore relevant to develop a fully automated method to assess preterm infant stages of sleep. This work is in progress.

The open-source software specifically designed for this study is also one important result of this study. ViSiAnnot was designed so that the user can easily configure the number of videos and signals to display, as well as the number of labels to annotate. Thus, it can adapt to other studies where it is needed to annotate temporal events. Multimodal visualization was necessary to get precise behavioral evaluation of sleep and to get a database that can be worked offline by different experts which gives strength to the annotations. To our knowledge there are only a very few software tools that allow multimodal visualization together with an annotation tool and none of them allow simultaneous visualization of video and physiological signals. ViSiAnnot will be available as an open-source software. The advantage is that the software will be available to everyone, not depending on a third-party software requiring a license and allowing collaborative work for improving the software and adding features.

Protecting the sleep periods of preterm infants is an important issue and is probably very important to promote optimal neurodevelopment of these vulnerable newborns. This study is a first step for the development of a method for QS detection and quantification in view to develop signal processing automation that will have to be compared with scoring using the neurophysiological methods. We expect that this will provide a useful and user-friendly, non-invasive, reproducible, continuous monitoring of sleep maturation for preterm infants that could be used as a decision support system for caregivers and clinicians in daily clinical practice.

## Data Availability Statement

The datasets presented in this article are not readily available because, the database generated and/or analyzed during the current study are not publicly available due to ethical issue. Requests to access the datasets should be directed to https://www.digi-newb.eu.

## Ethics Statement

The studies involving human participants were reviewed and approved by CPP Ouest IV 34/16. Written informed consent to participate in this study was provided by the participants' legal guardian/next of kin. Written informed consent was obtained from the minor(s)' legal guardian/next of kin for the publication of any potentially identifiable images or data included in this article.

## Author Contributions

LC, RW, SC, FP, and PP performed conceptualization. CF, J-MR, GF, GG, AT, and PP were responsible of the acquisitions. RW conceived the annotation tool. LC and PP performed recordings annotations. LC, FP, and ME performed the data analysis. LC, RW, FP, ME, and PP wrote the paper. LC, RW, FP, SC, ME, and PP participated to the review and editing process. All authors contributed to the article and approved the submitted version.

## Conflict of Interest

The authors declare that the research was conducted in the absence of any commercial or financial relationships that could be construed as a potential conflict of interest.

## Abbreviations

ADI: Average Duration of Intervals
AS: Active Sleep
ECG: Electrocardiogram
EEG: Electroencephalogram
GA: Gestational Age
IQR: Inter Quartile Range
NOK: Not OK intervals
PMA: Post-Menstrual Age
PPC: Personal Protection Committee
QS: Quiet Sleep
SS: Startles and Sighs
WA: Weeks of Amenorrhea.
